# High success rate of repeat colonoscopy with standard endoscopes in patients referred for prior incomplete colonoscopy

**DOI:** 10.1186/1471-230X-14-56

**Published:** 2014-03-29

**Authors:** Andrew J Gawron, Annapoorani Veerappan, Rajesh N Keswani

**Affiliations:** 1 Division of Gastroenterology and Hepatology, Feinberg School of Medicine, Northwestern University, Chicago, IL, USA; 2 Center for Healthcare Studies, Feinberg School of Medicine, Northwestern University, Chicago, IL, USA

**Keywords:** Incomplete colonoscopy, Enteroscopes, Endoscopy, Difficult colonoscopy

## Abstract

**Background:**

In patients with incomplete colonoscopy, cecal intubation is sometimes unsuccessful due to a redundant or tortuous colon. Repeat colonoscopy may be successful with the use of alternate endoscopes or careful attention to technique but limited outcomes data is available. The aim of this study was to describe the technique, success rate and outcomes of consecutive patients referred for previous incomplete colonoscopy.

**Methods:**

We conducted a retrospective chart review of incomplete colonoscopy procedures in patients age 18-90 at an academic teaching hospital referred to an endoscopist specializing in difficult colonoscopy.

**Results:**

Cecal intubation was successful in 96 of 100 repeat colonoscopies and 83 procedures were completed with a standard endoscope (adult, pediatric, or gastroscope). The adenoma detection rate was 28% for successful repeat colonoscopies; a majority of these patients had no adenomas identified on incomplete exam. In 69.4% of cases, an endoscope was used to successfully complete colonoscopy that was not used in the incomplete colonoscopy. The median insertion time was significantly less for the complete colonoscopy (10.6 min) compared to the incomplete colonoscopy (18.8 min, P = 0.004).

**Conclusions:**

Repeat colonoscopy has a high success rate and identified a significant number of new adenomas. Use of all available endoscopes should be considered prior to procedure termination in patients with a tortuous colon. Repeat colonoscopy can often be accomplished using a standard endoscope and is not attributed to increased endoscope insertion time.

## Background

Colonoscopy is a well-established procedure utilized for the evaluation of lower gastrointestinal tract diseases including the screening for colorectal polyps and cancer (CRC) [1]. Optical colonoscopy is performed via inserting a flexible tube retrograde through the rectum and the goal of a complete procedure is the advancement of the endoscope to the cecum. A recent study estimated that colonoscopy procedures have increased 50% over the past decade and over half of them were performed for CRC screening or surveillance [2]. Professional societies and The U.S. Multi Society Task Force on Colorectal Cancer targets a 95% completion rate for screening colonoscopies [3]. However, incomplete colonoscopy has been shown to occur in up to 13% of patients [4] and there are not clear guidelines regarding further management of these patients. Radiology has traditionally been used to facilitate complete colon evaluation in these patients, though barium studies are suboptimal in evaluating the colon for pathology [5]. CT colonography, while promising, has limitations in that it requires radiation exposure, may not detect flat polyps adequately, and is not widely available [6]. Additionally, any radiologic findings ultimately require subsequent endoscopic evaluation and/or removal.

There are a variety of factors that contribute to an incomplete colonoscopy including prior abdominal surgeries resulting in adhesions, severe diverticular disease, inadequate bowel cleansing, and patient discomfort [4,7]. Inadequate bowel cleansing can be corrected with an alteration in bowel preparation and patient discomfort can be addressed by modifying the anesthesia used. Difficult colon anatomy often requires the use of alternate techniques or screening modalities [8-17] when cecal intubation is unsuccessful due to a redundant (excessive looping) or tortuous (excessive angulation) colon. Endoscopic modalities include the use of smaller caliber colonoscopes, overtubes, fluoroscopy, or single and double balloon colonoscopy. There have been numerous studies reporting successful colonoscopy with specialty endoscopes in patients with a previous incomplete colonoscopy [11-16,18]. However, modalities used in clinical practice vary based upon the individual patient and are often limited based on available institutional expertise. We have recently shown that an incomplete colonoscopy referral program had only a modest impact on provider recommendations at our institution [19].

There is limited data on the outcomes of attempts at repeating colonoscopy with standard endoscopes after an incomplete procedure. Prior studies have shown that a variety of endoscopes and techniques can be used to achieve cecal intubation after prior incomplete colonoscopy but there has been little comparative data to the initial incomplete study [10,20,21]. In a randomized controlled trial comparing a standard endoscope (adult colonoscope) with a single balloon enteroscope, we reported a cecal intubation rate of 50% in the adult colonoscope arm [16]. However, we hypothesized that this success rate in the “standard” endoscope group would increase with a full complement (adult/pediatric colonoscope and upper endoscope) of endoscopes available. The objectives of this study were to describe the technique, success rate and outcomes of consecutive patients referred for repeat colonoscopy and compare endoscopes used and procedure time to the previous incomplete colonoscopy.

## Methods

### Study design

We conducted a retrospective chart review using administrative and manually extracted data at the Feinberg School of Medicine at Northwestern University. The Northwestern University Institutional Review Board approved the study.

### Inclusion criteria

Patients referred for repeat colonoscopy over a 26-month time period (April 2010 to May 2012) were eligible for the study. Patients with an incomplete colonoscopy due solely to inadequate preparation or sedation were excluded.

### Colonoscopy procedure

All incomplete procedures were performed using conscious sedation. All repeat procedures were performed by a single endoscopist (RK) using monitored anesthesia care. The choice of initial endoscope on repeat colonoscopy was not standardized. However, in general if the cause of the incomplete procedure was tortuousity (acute angulation of the colon), a pediatric colonoscope or upper endoscope was used. If the cause of the incomplete procedure was colon redundancy (elongation causing excessive looping), an adult colonoscope was used. The cause of the incomplete procedure as either due to colon tortuousity or redundancy was determined by review of the procedure report.

### Measures

Data extracted from the chart review included patient demographics (age, gender), BMI, history of prior surgeries, history of barium enemas, and prior incomplete colonoscopy characteristics (indication, number of prior colonoscopies, extent of prior colonoscopy, procedure duration, documented reasons for incomplete colonoscopy, and endoscopes used during the procedure). The same procedural measures were extracted for the repeat colonoscopy procedure. A “standard” endoscope was defined as an adult colonoscope (CF-H180AL), pediatric colonoscope (PCF-H180AL), or gastroscope (GIF-H180J, Olympus, Center Valley, PA). Adult and pediatric colonoscopes had variable stiffness capability allowing for adjustment of insertion tube flexibility and all endoscope had an auxiliary water channel. Pathology records were reviewed to determine the number of adenomas and cancers that were detected or removed at each procedure.

### Outcomes

The primary outcome was defined as the proportion of patients with a successful repeat complete colonoscopy after prior incomplete colonoscopy. Secondary outcomes included the number of times endoscopes were changed for each procedure (incomplete vs. complete), the proportion of patients requiring a different endoscope to complete the procedure, and procedure times (insertion, withdrawal, and total). Adenoma detection rates for incomplete and complete procedures are also reported.

### Statistical analyses

Descriptive statistics were calculated for each measure and are reported as medians (continuous variables) and proportions (categorical variables) of the total patient sample. The primary outcome is reported as a proportion of the total patient sample. Secondary outcomes are reported as proportions for categorical variables and medians for continuous variables. Differences in procedure times between the complete and incomplete procedures were determined using the Wilcoxon signed rank test. Median endoscope insertion times of documented redundant and tortuous colons were compared using the Wilcoxon rank-sum test. The difference in the proportion of type of endoscopes used to complete colonoscopy compared to endoscopes used for the prior incomplete colonoscopy was determined by a chi-squared test or Fisher’s exact test. All analyses were performed using STATA 12.0 (College Station, TX).

## Results

Over the study period, 32,246 colonoscopies were performed at our institution. A total of 100 patients with prior incomplete colonoscopy were referred to a single endoscopist for repeat colonoscopy attempt during the defined study time period. Patients were predominantly female (N = 67) with a median age of 62.9 yrs (range 23.7-93.0 yrs). The median BMI was 25.4 (range 15.9-52.1). A substantial minority of patients had a history of prior surgery as shown in Table 1, including open abdominal surgery (N = 18), pelvic/gynecologic surgery (N = 31), and laparoscopic abdominal surgery (N = 27).

**Table 1:** Patient sample and incomplete colonoscopy characteristics

	**N (Total = 100)**
**Patient characteristics**	
***Age (yrs), median (range)***	62.9 (23.7-93.0)
***Female sex***	67
***BMI, median (range)***	25.4 (15.9-52.1)
***History of surgery***	
Open abdominal surgery	18
Pelvic/gynecologic surgery	31
Laparoscopic abdominal surgery	27
**Incomplete colonoscopy characteristics**	
***Primary indication for colonoscopy***	
Screening	54
Surveillance (h/o polyps)	26
Other (symptoms, abnormal imaging)	20
***Number of prior incomplete colonoscopies***^***1***^	
1	80
2	11
4	1
***Extent of most recent colonoscopy***	
Left colon	41
Right colon	44
Documentation not available	15
***Documented primary reason for incomplete colonoscopy***	
Tortuosity	52
Redundancy	48
***Endoscopes used during incomplete colonoscopy***^***2***^	
Adult colonoscope	70 (82.4%)
Pediatric colonoscope	26 (30.6%)
EGD scope	4 (4.7%)
> 1 endoscope used	13 (15.3%)
***Adenoma’s detected/removed per procedure***	
0	75
1	6
2	2
3	1

^1^8 missing due to procedure being performed at other institutions.

^2^Percentages reflect total N = 85 with complete documentation.

Incomplete colonoscopy characteristics are also shown in Table 1. The majority of procedures were for a screening or surveillance indication. A total of 12 patients had more than one incomplete procedure with a single patient having 4 incomplete procedures prior to referral. The extent of the most recent incomplete procedure was to the left colon in 48.2% of patients and right colon in 51.8% patients in those patients with a documented extent (N = 85). Incomplete procedures were attributed to tortuosity (N = 52, 61.2%) and redundancy (N = 48, 56.5%). Adult colonoscopes were used in the majority of cases (N = 70) with a lesser amount of pediatric (N = 26) and gastroscopes (N = 4) used for the procedures. Multiple endoscopes were used in a minority (13%) of incomplete procedures. Only an adult colonoscope (without switching to pediatric or upper endoscope) was used in 25% of incomplete procedures due to a tortuous colon.

**Figure 1:**
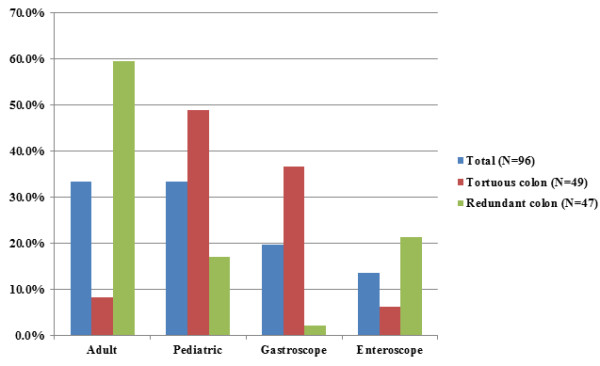
Endoscope type used to achieve complete colonoscopy.

**Figure 2:**
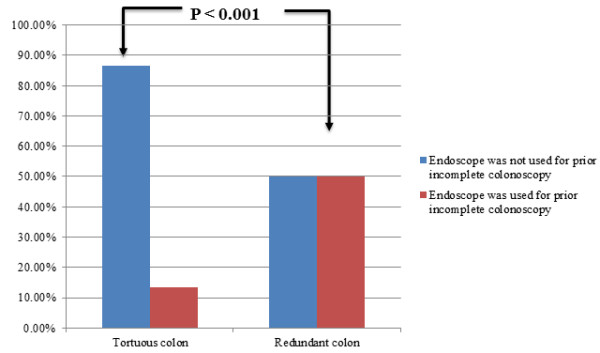
Endoscopes used to complete colonoscopy compared to endoscopes used for prior incomplete colonoscopy.

The primary outcome of successful cecal intubation rate was 96% for repeat colonoscopy (Table 2) and this was achieved with a standard endoscope in the majority of cases (83%). The majority of patients (71.8%, N = 61) underwent repeat exam within 1 year of incomplete colonoscopy; the median time from incomplete to repeat colonoscopy was 85.3 days (IQR 586.6 days). There were no procedural complications among the study sample. Cecal intubation was unsuccessful in 4 patients (4%) due to inguinal hernia (N = 1), Crohn’s disease stricture (N = 1), redundancy (N = 1), and tortuosity (N = 1). As shown in Figure 1, for the entire patient sample with a complete procedure, cecal intubation was achieved with an adult colonoscope (N = 32), pediatric colonoscope (N = 32), gastroscope (N = 19), or single balloon enteroscope (N = 13). A total of 59 procedures (69.4%) required an endoscope to successfully complete colonoscopy that was not used in the prior incomplete colonoscopy, including balloon enteroscopes. In patients with an incomplete colonoscopy attributed to a tortuous colon, successful colonoscopy was completed in the majority of patients using a standard smaller caliber endoscope (pediatric colonoscope or gastroscope) not used in the initial procedure (Figure 1). In the 17 patients requiring an enteroscope or who had an otherwise unsuccessful repeat procedure, the reason for the prior incomplete procedure was more often due to redundancy (64.7% vs. 44.6%), although this did not reach statistical significance (P = 0.1). Patients with a documented tortuous colon required an endoscope that was not used for the previous incomplete colonoscopy 86.0% of the time compared to 50% of the time in patients with a redundant colon (Figure 2, chi-squared *P* < 0.001).

**Table 2:** Repeat colonoscopy characteristics (after incomplete colonoscopy)

	**N (Total = 100)**
***Extent of colonoscopy***	
Incomplete	4
Complete (cecum, terminal ileum)	96
***Time duration from incomplete to repeat colonoscopy***	
< 1 year	61
= or > 1 year	22
Unknown	17
***Time between procedures (incomplete to repeat)***	
Median days (IQR)	85.3 (586.6)
***Endoscopes used during procedures***^***1***^	
Adult colonoscope	35 (35%)
Pediatric colonoscope	43 (43%)
EGD scope	23 (23%)
Enteroscope	14 (14%)
> 1 endoscope	15 (15%)
***Adenoma’s detected/removed***	
0	72
1	19
2	7
3	1
10	1
***Adenocarcinoma detected***	1

^1^Total >100 due to procedures requiring more than one endoscope during procedures.

The adenoma detection rate was 9% for all incomplete procedures. In contrast, the overall adenoma detection rate was 28% (range 1-10 adenomas) for successful repeat colonoscopies. In the subgroup of patients with repeat colonoscopy within 1 year of incomplete exam, the adenoma detection rate was 31.7%. Among these patients, 89.5% had no adenomas on initial incomplete colonoscopy. Of note, a single cecal adenocarcinoma was detected in a patient with an unremarkable barium study 2 days prior to the exam.

For patients in whom complete time data was available for both procedures the median insertion time was significant less for the complete colonoscopy (10.6 min) compared to the incomplete colonoscopy (18.8 min, *P* = 0.004 in patients with matched time data) [Table 3]. The total procedure times for incomplete and complete procedures were 20.8 min and 22.7 min, respectively (*P* = 0.06 in patients with matched time data). There was not a statistically significant difference in insertion times for complete colonoscopy when comparing patients with documented tortuous (N = 41, median time 9.4 min, IQR 11.15) or redundant (N = 35, median time 11.0 min, IQR 7.8) colons (*P* = 0.1).

**Table 3:** Procedure times for incomplete and complete colonoscopies

	**Incomplete colonoscopy**		**Complete colonoscopy**		***P*** **value**^**1**^
***Procedure time***	N^2^	Median (range)	N^2^	Median (range)	
Insertion time (min)	28	18.8 (8.2-42.2)	89	10.6 (0.4-135.6)	0.004^3^
Withdrawal time (min)	26	3.6 (0.02-20.5)	83	10.0 (1.0-43.9)	0.002^4^
Total procedure time (min)	52	20.8 (1.9-133.3)	82	22.7 (7.7-148.9)	0.06^5^

^1^Wilcoxon signed rank test comparing only those patients with available time data for both incomplete and complete procedures.

^2^Number of patients for which time data was available from the electronic medical record.

^3^N = 24.

^4^N = 20.

^5^N = 44.

## Discussion

Our results show that in patients referred for incomplete colonoscopy, a complete exam was achieved in 96% of patients and with a standard endoscope in 83% of cases. A sizable number of adenomas were found on repeat procedure. Furthermore, a majority of patients required use of an endoscope that was not used in the prior incomplete procedure. In particular, patients with a tortuous colon more often had a repeat complete procedure with a standard endoscope that was not used in the prior incomplete procedure. Our data also suggest that incomplete procedures due to colonic redundancy may more often require referral for use of balloon enteroscopes to complete the procedure.

While incomplete studies due to inadequate bowel preparation or sedation can be rectified with modified sedation or bowel purgative, incomplete studies due to colon redundancy or tortuosity may be more challenging to manage. We and others have shown that repeat colonoscopy is successful in a large majority of patients referred for prior incomplete procedures utilizing specialty endoscopes [11-15,18]. A limitation of these studies is that they often rely exclusively on studying specialty endoscopes whereas in daily practice there are a full range of other standard endoscopes which may facilitate cecal intubation with careful attention to technique. Our results suggest that many incomplete colonoscopies can be completed with standard endoscopes, especially in patients with tortuous colons.

Two large retrospective studies, both from the same endoscopist, have been published with similar findings [10,20]. In these studies, a wide variety of endoscopes and techniques were used to facilitate cecal intubation after prior incomplete colonoscopy. As in our study, cecal intubation was successful in nearly all patients (96%). However, our study is different in that complete colonoscopy was achieved mostly using standard endoscopes, which are more readily available than specialty scopes. Also, prior studies provided minimal data on the initial incomplete colonoscopy and, thus, an understanding of what differences facilitated cecal intubation was not possible. Our data should encourage endoscopists that many difficult colonoscopies can likely be completed with standard endoscopes before subjecting patients to a referral for a second procedure which carries added cost and additional risk. We have demonstrated that repeat colonoscopy does not require significantly greater endoscope insertion time. However, the majority of patients did require use of an endoscope not used during prior incomplete colonoscopy suggesting that failure to switch to another available endoscope may be an important contributing factor to incomplete colonoscopy. In addition, procedures with a tortuous colon may be completed more often by switching to a different smaller caliber standard endoscope.

There are several limitations to our study that merit attention. Our study represents the experience of a referral based program at a high volume tertiary academic enter and further work is needed to determine if the results are generalizable to other practice settings and individual endoscopists. This study was not a randomized controlled study; as this was a retrospective study using clinical data, complete information was not available on certain procedures including the use of ancillary techniques (water immersion, patient positioning, or external pressure). Furthermore, the choice of endoscope used was determined by endoscopist preference and not by a predetermined protocol. This study also represents the efforts of a single endoscopist which may not be generalizable to all endoscopists. Finally, all procedures in this study were performed with monitored anesthesia care. While this may be unnecessary in many cases, it is used routinely at our institution for complex endoscopy and we have found its use eliminates the possibility of patient discomfort limiting colonoscopy completion in select cases.

## Conclusions

**Figure 3:**
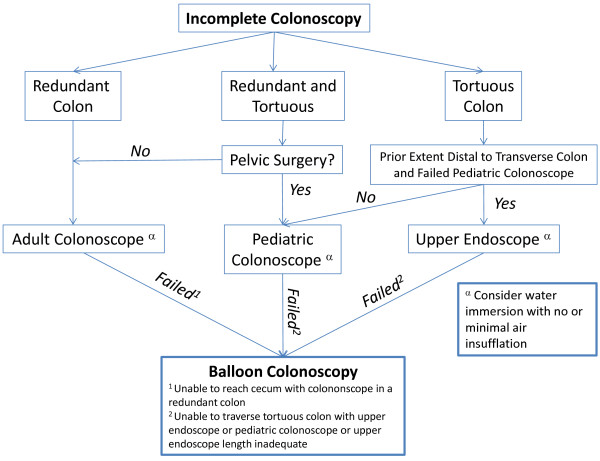
Proposed approach to patients with incomplete colonoscopy.

In summary, repeat colonoscopy is successful in the vast majority of patients referred for prior incomplete studies. Furthermore, repeat colonoscopy does not appear to take substantially more time to complete and often can be completed using a standard endoscope. Larger, prospective studies are warranted to confirm these findings and these are ongoing. Based on the current data, we would recommend that practitioners attempt to use a smaller caliber endoscope when encountering a tortuous colon prior to prematurely terminating the procedure. Our proposed approach to patients with incomplete colonoscopy is shown in Figure 3; prospective studies are underway to determine the validity of this approach. Additionally, persistence and adherence to good technique may facilitate a complete exam when encountering colonic redundancy but practitioners should be aware that some patients may need referral for the use of specialized enteroscopes to complete the procedure. Institutions should work towards establishing an incomplete colonoscopy referral program to facilitate complete colon evaluation in patients with prior incomplete studies due to difficult anatomy.

## Abbreviations

CRC: Colorectal cancer; IQR: Interquartile range.

## Competing interest

The authors declare that they have no competing interest.

## Authors’ contributions

AJG: Analysis and Interpretation of Data, Writing of Manuscript. AV: Data Acquisition. RNK: Study Conception and Design, Analysis and Interpretation of Data, Writing of Manuscript. All authors read and approved the final manuscript.

## Pre-publication history

The pre-publication history for this paper can be accessed here:

http://www.biomedcentral.com/1471-230X/14/56/prepub

## References

[B1] Lieberman DA Rex DK Winawer SJ Giardiello FM Johnson DA Levin TR Guidelines for colonoscopy surveillance after screening and polypectomy: a consensus update by the US Multi-Society Task Force on Colorectal Cancer Gastroenterol 2012 143 844 857 10.1053/j.gastro.2012.06.001 22763141

[B2] Peery AF Dellon ES Lund J Crockett SD McGowan CE Bulsiewicz WJ Gangarosa LM Thiny MT Stizenberg K Morgan DR Ringle Y Kim HP Dibonaventura MD Carroll CF Allen JK Cook SF Sandler RS Kappelman MD Shaheen NJ Burden of gastrointestinal disease in the United States: 2012 update Gastroenterol 2012 143 1179 1187 e1171-1173 10.1053/j.gastro.2012.08.002 PMC3480553 22885331

[B3] Rex DK Petrini JL Baron TH Chak A Cohen J Deal SE Hoffman B Jacobson BC Mergener K Petersen BT Safdi MA Faigel DO Pike IM Quality indicators for colonoscopy Am J Gastroenterol 2006 101 873 885 16635231 10.1111/j.1572-0241.2006.00673.x

[B4] Shah HA Paszat LF Saskin R Stukel TA Rabeneck L Factors associated with incomplete colonoscopy: a population-based study Gastroenterol 2007 132 2297 2303 10.1053/j.gastro.2007.03.032 17570204

[B5] Kao KT Tam M Sekhon H Wijeratne R Haigh PI Abbas MA Should barium enema be the next step following an incomplete colonoscopy? In J Colorectal Dis 2010 25 1353 1357 10.1007/s00384-010-1014-6 20652709

[B6] Sakamoto T Mitsuzaki K Utsunomiya D Matsuda K Yamamura S Urata J Kawakami M Yamashita Y Detection of flat colorectal polyps at screening CT colonography in comparison with conventional polypoid lesions Acta Radiol 2012 53 714 719 10.1258/ar.2012.110685 22821957

[B7] Neerincx M Droste JS T s Mulder CJ Rakers M Bartelsman JF Loffeld RJ Tuynman HA Brohet RM van der Hulst RW Colonic work-up after incomplete colonoscopy: significant new findings during follow-up Endoscopy 2010 42 730 735 10.1055/s-0030-1255523 20669092

[B8] Morini S Zullo A Hassan C Lorenzetti R Campo SM Endoscopic management of failed colonoscopy in clinical practice: to change endoscopist, instrument, or both? In J Colorectal Dis 2011 26 103 108 10.1007/s00384-010-1016-4 20686778

[B9] Hanson ME Pickhardt PJ Kim DH Pfau PR Anatomic factors predictive of incomplete colonoscopy based on findings at CT colonography AJR Am J Roentgenol 2007 189 774 779 10.2214/AJR.07.2048 17885044

[B10] Rex DK Chen SC Overhiser AJ Colonoscopy technique in consecutive patients referred for prior incomplete colonoscopy Clin Gastroenterol Hepatol 2007 5 879 883 10.1016/j.cgh.2007.03.015 17544873

[B11] Kaltenbach T Soetikno R Friedland S Use of a double balloon enteroscope facilitates caecal intubation after incomplete colonoscopy with a standard colonoscope Dig Liv Dis 2006 38 921 925 10.1016/j.dld.2006.08.003 16990055

[B12] Pasha SF Harrison ME Das A Corrado CM Arnell KN Leighton JA Utility of double-balloon colonoscopy for completion of colon examination after incomplete colonoscopy with conventional colonoscope Gastrointest Endosc 2007 65 848 853 10.1016/j.gie.2006.08.046 17324408

[B13] Moreels TG Macken EJ Roth B Van Outryve MJ Pelckmans PA Cecal intubation rate with the double-balloon endoscope after incomplete conventional colonoscopy: a study in 45 patients J Gastroenterol Hepatol 2010 25 80 83 10.1111/j.1440-1746.2009.05942.x 19686405

[B14] Suzuki T Matsushima M Tsukune Y Fujisawa M Yazaki T Uchida T Gocyo S Okita I Shirakura K Sasao K Salto T Sakamoto I Igarashi M Koike J Takagi A Mine T Double-balloon endoscopy versus magnet-imaging enhanced colonoscopy for difficult colonoscopies, a randomized study Endoscopy 2012 44 38 42 22143991 10.1055/s-0030-1256875

[B15] Hotta K Katsuki S Ohata K Abe T Endo M Shimatani M Nagaya T Kusaka T Matsuda T Uraoka T Yamaguchi Y Murakami Y Salto Y A multicenter, prospective trial of total colonoscopy using a short double-balloon endoscope in patients with previous incomplete colonoscopy Gastrointest Endosc 2012 75 813 818 10.1016/j.gie.2011.11.020 22284085

[B16] Keswani RN Single-balloon colonoscopy versus repeat standard colonoscopy for previous incomplete colonoscopy: a randomized, controlled trial Gastrointest Endosc 2011 73 507 512 10.1016/j.gie.2010.09.047 21145054

[B17] Dzeletovic I Harrison ME Pasha SF Crowell MD Decker GA Gurudu SR Leighton JA Comparison of single- versus double-balloon assisted-colonoscopy for colon examination after previous incomplete standard colonoscopy Dig Dis Sci 2012 57 2680 2686 10.1007/s10620-012-2227-z 22615017

[B18] Schembre DB Ross AS Gluck MN Brandabur JJ McCormick SE Lin OS Spiral overtube-assisted colonoscopy after incomplete colonoscopy in the redundant colon Gastrointest Endosc 2011 73 515 519 10.1016/j.gie.2010.11.047 21353848

[B19] Gawron AJ Veerappan A McCarthy ST Kankanala V Keswani RN Impact of an incomplete colonoscopy referral program on recommendations after incomplete colonoscopy Dig Dis Sci 2013 58 1849 1855 10.1007/s10620-013-2605-1 23456503

[B20] Vemulapalli KC Rex DK Water immersion simplifies cecal intubation in patients with redundant colons and previous incomplete colonoscopies Gastrointest Endosc 2012 76 812 817 10.1016/j.gie.2012.05.033 22901988

[B21] Brahmania M Park J Svarta S Tong J Kwok R Enns R Incomplete colonoscopy: maximizing completion rates of gastroenterologists Can J Gastroentrol 2012 26 589 592 10.1155/2012/353457 PMC3441163 22993727

